# The transcription factor *dfoxo* controls the expression of insulin pathway genes and lipids content under heat stress in *Drosophila melanogaster*

**DOI:** 10.18699/VJ21053

**Published:** 2021-09

**Authors:** M.A. Eremina, P.N. Menshanov, O.D. Shishkina, N.E. Gruntenko

**Affiliations:** Institute of Cytology and Genetics of the Siberian Branch of the Russian Academy of Sciences, Novosibirsk, Russia; Institute of Cytology and Genetics of the Siberian Branch of the Russian Academy of Sciences, Novosibirsk, Russia Novosibirsk State Technical University, Novosibirsk, Russia; Institute of Cytology and Genetics of the Siberian Branch of the Russian Academy of Sciences, Novosibirsk, Russia; Institute of Cytology and Genetics of the Siberian Branch of the Russian Academy of Sciences, Novosibirsk, Russia

**Keywords:** *Drosophila melanogaster*, insulin/insulin-like growth factors signaling pathway, dInR, *dilp6*, *dfoxo*, gene-expression, feeding behaviour, total lipids, *Drosophila melanogaster*, сигнальный каскад инсулина/инсулиноподобных факторов роста, dInR, *dilp6*, *dfoxo*, экспрессия генов, пищевое поведение, общие липиды

## Abstract

The insulin/insulin-like growth factor signaling (IIS) pathway is one of the key elements in an organism’s response to unfavourable conditions. The deep homology of this pathway and its evolutionary conservative role in controlling the carbohydrate and lipid metabolism make it possible to use *Drosophila melanogaster* for studying its functioning. To identify the properties of interaction of two key IIS pathway components under heat stress in *D. melanogaster* (the forkhead box O transcription factor (*dfoxo*) and insulin-like peptide 6 (*dilp6*), which intermediates the *dfoxo* signal sent from the fat body to the insulin-producing cells of the brain where DILPs1–5 are synthesized), we analysed the expression of the genes *dilp6*, *dfoxo* and insulin-like receptor gene *(dInR)* in females of strains carrying the hypomorphic mutation *dilp6*^41^and hypofunctional mutation *foxo*^BG01018^. We found that neither mutation inf luenced *dfoxo* expression and its uprise under short-term heat stress, but both of them disrupted the stress response of the *dilp6* and dInR genes. To reveal the role of identif ied disruptions in metabolism control and feeding behaviour, we analysed the effect of the *dilp6*^41^ and *foxo*^BG01018^ mutations on total lipids content and capillary feeding intensity in imago under normal conditions and under short-term heat stress. Both mutations caused an increase in these parameters under normal conditions and prevented decrease in total lipids content following heat stress observed in the control strain. In mutants, feeding intensity was increased under normal conditions; and decreased following short-term heat stress in all studied strains for the f irst 24 h of observation, and in *dilp6*^41^ strain, for 48 h. Thus, we may conclude that *dfoxo* takes part in regulating the IIS pathway response to heat stress as well as the changes in lipids content caused by heat stress, and this regulation is mediated by *dilp6*. At the same time, the feeding behaviour of imago might be controlled by *dfoxo* and *dilp6* under normal conditions, but not under heat stress.

## Abstract (ru)

Одним из основных элементов ответа организма на неблагоприятные условия является сиг- нальный каскад инсулина/инсулиноподобных факторов роста (И/ИФР). Благодаря глубокой гомологии этого каскада и эволюционной консервативности его роли в регуляции углеводно-жирового метаболизма, воз- можно использование модельного объекта *Drosophila melanogaster* для изучения механизмов его функционирования. Для определения особенностей взаимодействия двух ключевых компонентов каскада И/ИФР у *D. melanogaster* – транскрипционного фактора *dfoxo* и инсулиноподобного пептида *dilp6*, «посредника» в передаче сигнала от *dfoxo* в жировом теле к инсулин-продуцирующим клеткам мозга (месту синтеза DILPs1–5), в условиях теплового стресса мы провели анализ экспрессии генов *dilp6*, *dfoxo* и гена добного рецептора *(dInR)* у самок линий, несущих гипоморфную мутацию *dilp6*^41^и гипофункциональную му- тацию *foxo*^BG01018^. Обнаружено, что обе мутации не оказывали влияния на экспрессию *dfoxo* и ее повышение при кратковременном тепловом стрессе, однако нарушали ответ на стресс генов *dilp6* и dInR. Для выявления роли обнаруженных нарушений в контроле метаболизма и метаболического поведения мы проанализиро- вали влияние мутаций *dilp6*^41^и *foxo*^BG01018^ на содержание общих липидов и интенсивность капиллярного пи- тания имаго в нормальных условиях и при кратковременном тепловом стрессе. Обе мутации приводили к усилению данных признаков в нормальных условиях и препятствовали снижению содержания общих липи- дов после стресса, наблюдаемому у контрольной линии. Интенсивность питания была повышена у мутантов в нормальных условиях и снижалась после кратковременного теплового стресса у всех изученных линий в течение первых суток наблюдения, а у линии *dilp6*^41^– в течение двух суток. Таким образом, можно заключить, что *dfoxo* принимает участие в регуляции как ответа сигнального каскада И/ИФР на тепловой стресс, так и вызываемых тепловым стрессом изменений в содержании липидов, причем эта регуляция опосредуется *dilp6*. В то же время метаболическое поведение имаго, по-видимому, регулируется *dfoxo* и *dilp6* в нор- мальных условиях, но не при тепловом стрессе.

## Introduction

Nowadays, as living beings often encounter unfavourable environmental conditions such as pollution and global warming, the study of deeply conservative mechanisms that contribute to adaptation is of current interest. It is known that such influences launch the development of nonspecific adaptive defensive responses on molecular (Garbuz, Evgen’ev, 2017), behavioral (Kaluev, 1999), biochemical and physiological (Gruntenko, 2008; Even et al., 2012; Miyashita, Adamo, 2020) levels. The ability to respond to stress in an integrated manner, which comprises behavioral, metabolic and molecular reactions, is key for survival and adaptation of animals including insects (Koyama et al., 2020). It was proven that besides its role as crucial modulator of growth and metabolism, in insects, the IIS pathway is an essential component of the neuroendocrine stress reaction (Gruntenko, Rauschenbach, 2018; Lubawy et al., 2020). Due to the deep homology of this pathway in animals of different taxa including humans and flies, it is possible to use the latter as an object for investigating evolutionary-conservative mechanisms underlying molecular- genetic regulation of the IIS pathway, and carbohydrate and lipid metabolism it controls. As in most animals, in insects, carbohydrates and lipids serve as the main energy supply (Arrese, Soulages, 2010). The processes of producing and storing energy undergo complex modulation by many inner factors including heritage, lifestyle, hormones, metabolites, as well as various outside influences (Mattila, Hietakangas, 2017).

**Fig. 1.**
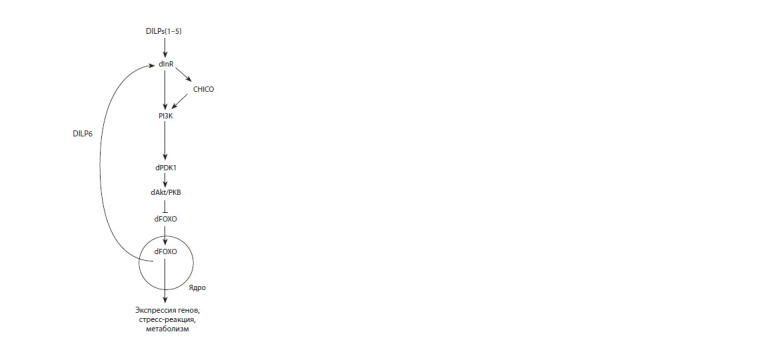
The scheme of insulin/insulin-like growth factors signaling pathway in Drosophila DILPs are Drosophila insulin-like growth peptides, dInR – Drosophila insulinlike receptor, CHICO – homologue of mammalian insulin receptor substrate, PI3K – phosphoinositide 3 kinase, dPDK1 – Drosophila phosphoinositidedependent kinase-1, dAkt/PKB – a homolog of mammalian protein kinase B, dFOXO – Drosophila forkhead box O transcription factor.

*Drosophila’s*applicability to the research of metabolism is defined by the simplicity of its IIS pathway regulation (Fig. 1), which involves homologues of insulin (DILPs1–5) and insulin-like growth factors (*dilp6*) of mammals connecting to a single insulin-like receptor *(dInR)*, which activates the pathway (Gruntenko, Rauschenbach, 2018), and two homologues of relaxin (DILPs7,8) (Gontijo, Garelli, 2018). The dInR signal being transduced directly or *via* its substrate CHICO (the homologue of insulin receptor substrates of mammals, IRS1–4) causes dAkt/PKB (protein kinase В homologue) to activate, which in turn modulates the activity of a number of proteins, in particular, it phosphorylates transcriptional factor of Forkhead box class O family, *dfoxo* (homologue of mammalian *foxo*), which is synthesized in the fat body and controls the transcription of more than a thousand genes (Bai et al., 2012), and inhibits its translocation into the nucleus (Puig et al., 2003; Slack et al., 2011; Álvarez-Rendón et al., 2018). Under stress, *dfoxo* is translocated to the nucleus (Jünger et al., 2003; Hwangbo et al., 2004; Gruntenko et al., 2016) activating the expression of a number of genes including dInR *via* a feedback loop (Gruntenko, Rauschenbach, 2018). It was also previously shown that the expression of *dilp6* in the fat body inhibits the expression of *dilp2* and *dilp5* in imago’s brain as well as the secretion of DILP2 into the hemolymph, and that *dfoxo* influence on the expression of DILPs produced in median neurosecretory cells is mediated by *dilp6* synthesized in the fat body (Slaidina et al., 2009; Bai et al., 2012). Thus, DILPs seems to connect *dfoxo*, adipose tissue and endocrine function of the brain, creating a feedback loop back to dInR.

DILPs are Drosophila insulin-like growth peptides, dInR – Drosophila insulinlike receptor, CHICO – homologue of mammalian insulin receptor substrate, PI3K – phosphoinositide 3 kinase, dPDK1 – Drosophila phosphoinositidedependent kinase-1, dAkt/PKB – a homolog of mammalian protein kinase B, *dfoxo* – Drosophila forkhead box O transcription factor.

Stress reaction causes the mobilization of organism’s energy reserves along with a variety of metabolic changes. In a changing environment, feeding behaviour plays an important role in adaptation (Rabasa, Dickson, 2016). It is known that in mammals, acute stress is usually accompanied by feeding suppression and a decrease in weight gain; chronic stress can result in excessive food intake, weight gain and obesity (Rabasa, Dickson, 2016).

This study aimed to analyse the expression of dInR, *dilp6* and *dfoxo* genes of three key components of the IIS pathway, which is involved in neuroendocrine stress reaction, in *D. melanogaster* strains carrying *dilp6*^41^ и *foxo* ^BG01018^ mutations under heat stress, and to evaluate the latter’s influence on feeding behaviour and total lipids content in these strains.

## Materials and methods

*Drosophila* strains and stress conditions. Three *D. melanogaster* strains were used in this study: strain *dilp6 ^41^* with the deletion covering the 3′ region of phl gene and 5′ upstream region of *dilp6* including the first exon and part of the first intron (Rauschenbach et al., 2017); strain *foxo* ^BG01018^, which carries a P[GT1] element transposon in the 5′ upstream region of the *dfoxo* gene, resulting in a mild loss of function (Dionne et al., 2006); and their progenitor strain w1118 as a control. The stocks were obtained from the Bloomington Drosophila Stock Center (Bloomington, IN, USA).

The cultures were raised on standard medium (agar-agar, 7 g/l; corn grits, 50 g/l; dry yeast, 18 g/l; sugar, 40 g/l) and kept at 25 °C, 12:12 h photoperiod, relative humidity 50 %. Imagoes were synchronised at eclosion (flies were collected every 3–4 hours). Females were exposed to heat stress by transferring vials with flies from a 25 °C incubator to a 38 °C incubator for 60 or 90 min. After 60 min of stressing flies were returned to 25 °C, after 90 min they were subsequently frozen in liquid nitrogen and stored at –80 °C.

**Quantitative real-time polymerase chain reaction (qRT-PCR).** mRNA quantity of *dilp6*, *dfoxo* and dInR genes was evaluated in whole body homogenates of Canton-S females (15 flies/sample) using TRI reagent Lot #BCBT8883 (Sigma-Aldrich, USA) for total RNA extraction, Revert Aid First Strand cDNA Synthesis Kit #K1621 (Thermo Fisher Scientific, USA) with oligo (dT)18 primer for synthesis of cDNA, M-427 Kit with SYBR-Green I (Syntol, Russia) and CFX96 Touch qPCR System (Bio-Rad, USA) for performing qRT-PCR. Each reaction was performed in triplicates with three biological replicates. Data were normalized against Act5C. High stability of Act5C expression under heat stress was shown by Ponton et al. (2011). The primers used in the study are shown in the Table.

**Tab:**
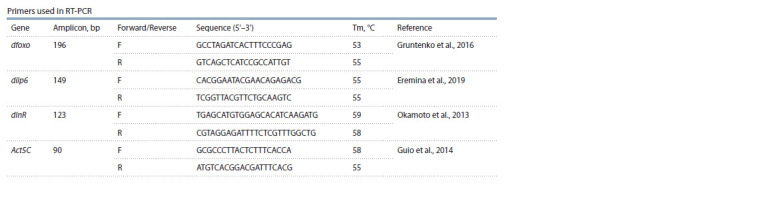
Primers used in RT-PCR

**Total lipid quantification.** Quantification of total lipids was performed using Van Handel’s method (1985) modified for *D. melanogaster* (Eremina, Gruntenko, 2020) under normal conditions or in 24 h after 60 min under 38 °С. Flies (1 fly per sample, 10–20 samples per each studied group) were decapitated to avoid the influence of eye pigment on the measurement results, homogenised on ice in 100 μl of chloroformmethanol (1:1) and shacked for 10 min. 50 μl of supernatant were transferred to new tubes and placed in microthermostat M-208 (Bis-N, Russia) at 90 °С till the solvent completely evaporated. Then 10 μl of 95 % H2SO4 were added to each sample and they were again kept at 90 °С for 2 min. After that the samples were cooled on ice and the phosphovanillin color reagent (85 % H3PO4 + 6 % vanillin solution (4:1)) was added up to 1 ml of volume. The samples were incubated for 15 min at room temperature till pink colouration appeared and was stable for 1 h. Then the samples were measured by Smart Spec Plus spectrophotometer (Bio-Rad, USA) at 525 nm.

**Feeding behaviour analysis (CAFE)** Ingestion was measured using the Capillary Feeder (CAFÉ) method of Ja et al. (2007), modified by Williams et al. (2014). To provide flies with a humid environment, flat-bottomed glass vials (20×100 mm) with 1 % agarose (5 cm high) were placed into microcentrifuged 50 ml tubes filled with 7 ml of water. Each glass capillary (10 × 90 mm, Narishige, Japan) was filled with 20 μl of liquid food containing 5 % sugar and 5 % east extract (Biospringer, France). Five females were placed into each vial (4–9 vials per group), which was plugged with a foam plug. A capillary was inserted into it through 10 μl and 200 μl pipette tips and was held in place by them. The vials with flies were kept in an incubator (Sanyo, Japan) at 25 °C, 50 % relative humidity, 12:12 h photoperiod for 24 or 48 h. Before that the experimental group was subjected to short-term heat stress (38 °C, 60 min). Initial and final food levels in capillaries were marked to determine total food consumption per day. To minimize food evaporation, capillaries were topped with a 0.1 μl oil layer. To adjust for food evaporation, a vial without flies was used.

**Statistical analysis.** Data on gene expression were analyzed by the 2^−ΔΔCT^ method (Livak, Schmittgen, 2001). All data are presented as means ± SEM and analysed by ANOVA. The results were considered significant at p < 0.05.

## Results and discussion

**Fig. 2.**
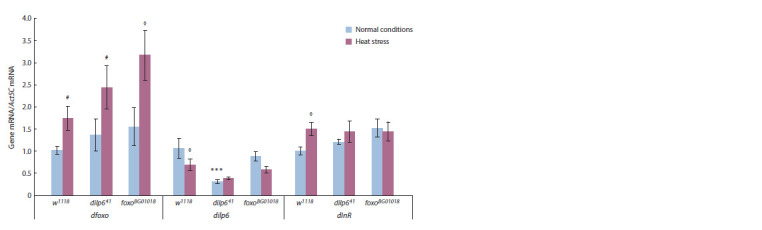
*dilp6*, *d*foxo** and dInR mRNA levels in *D. melanogaster*females of w1118, *dilp6*^41^and *foxo*^BG01018^ strains under normal conditions and after short-term heat stress (38 °С, 90 min). Each value is a mean of three biological replicates. Error bars show standard error of the mean. Asterisks indicate significant differences between females with mutation of Drosophila insulin-like peptide 6 gene (dilp641) and females of the control strain w1118 ( p < 0.001). Diamond indicates significant differences between stressed and control groups of the same genotype ( p < 0.05). Hash indicates a tendency for such differences ( p <0.07).

To discover whether disruption of the feedback loop of the IIS pathway regulation affects its stress response, we studied the expression of three key genes of the pathway, *dilp6*, *dfoxo* and dInR, in *D. melanogaster* females carrying hypomorphic mutation *dilp6 ^41^*and hypofunctional mutation *foxo* ^BG01018^ under normal conditions or heat stress (38 °С, 90 min). There were no quantitative changes in mRNA expression level of *dilp6* and dInR genes in *dilp6* ^41^and *foxo* ^BG01018^ strains under heat stress, whereas in their progenitor strain w1118 the expression of *dilp6* decreased, and the expression of dInR increased under heat stress (Fig. 2, p <0.05 for both genes). At the same, *dfoxo* expression level increased or had a tendency to increase under heat stress in all strains under study (see Fig. 2, STRAIN – F(2, 12)= 3.14, p <0.081; STRESS – F(1, 12)= 12.80, p < 0.0038). Notably, *dilp6* ^41^mutants are characterised by a lower *dilp6* expression ( p < 0.001); however, *dfoxo* expression in *foxo* ^BG01018^ mutants does not differ from the control strain w1118 (see Fig. 2). This allows us to assume that the previously described loss of *dfoxo* function in *foxo* ^BG01018^ strain (Dionne et al., 2006) is connected not with a lowered expression level of the corresponding gene but with a defect in its structure.

**Fig. 3.**
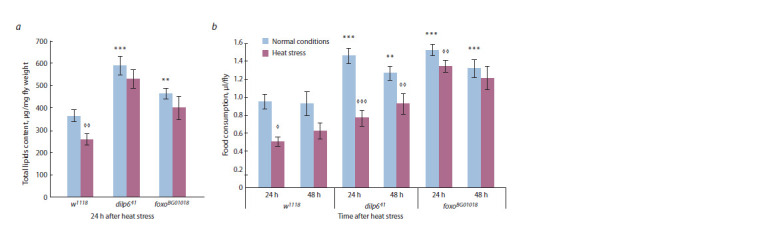
Total lipids level (a) and capillary feeding intensity (b) in females of *D. melanogaster *strains w1118, *dilp6*^41^and *foxo*^BG01018^ under normal conditions and following short-term heat stress (38 °С, 60 min). Each value is a mean of 10–20 (a) and 9–11 (b) measurements. Error bars indicate s.e.m. Asterisk indicates signif icant differences between control females of w1118 strain and females with dilp641 and foxoBG01018 mutations (** p < 0.01, *** p < 0.001). Diamond indicates signif icant differences between stressed and control groups of the same genotype (◊ – p < 0.05, ◊ ◊ – p < 0.01, ◊ ◊ ◊ – p < 0.001).

The results of qualitative measurement of total lipids in *D. melanogaster*females with *dilp6* ^41^and *foxo* ^BG01018^ mutations under normal conditions or following heat stress (38 °С, 60 min) signify that both mutations cause an increase in lipid content in comparison with the control strain w1118, and lipid content in *dilp6* ^41^and *foxo* ^BG01018^ strains, unlike in their progenitor strain, does not decrease in 24 h after heat stress(Fig. 3, a, STRAIN – F(1, 96) = 26.78, p ≪ 0.0001; STRESS – F(1, 96) = 1^41^.56, p <0.012; STRAIN*STRESS – F(2, 96) = = 0.25, p = 0.777).

The increased lipid content in females of the mutant strains could be explained by their discovered increased food consumption in comparison with control females of w1118 strain throughout the entire experiment (see Fig. 3, b, STRAIN – F(2, 59) = 44.40, p ≪ 0.0001; TIME – F(1, 59) = 5.12, p < 0.028; STRAIN*TIME – F(2, 59) = 1.^41^, p = 0.252). However, in the first 24 h after heat stress feeding intensity decreases in comparison with normal conditions in both females of the control strain w1118 and the mutant strains; in *dilp6* ^41^strain, this effect is maintained for 48 h (see Fig. 3, b, STRESS – F(1, 59) = 36.09, p ≪ 0.0001; STRAIN*STRESS – F(2, 59) = 6.28, p < 0.0034; STRAIN*STRESS*TIME – F(2, 59) = 1.26, p = 0.291).

It was previously shown that the IIS pathway can interact with gonadotropins and biogenic amines in Drosophila modulating their dynamics under stress and thus participating in the control of organism’s stress response (Gruntenko, Rauschenbach, 2018). However, it remained unclear (1) which links of the IIS pathway were involved in stress response and (2) what effect does the participation of the IIS pathway in stress response have on its ability to control the carbohydrate and lipid metabolism

It was demonstrated by us earlier that in *D. melanogaster* females *dfoxo* translocates to the nucleus under heat stress (Gruntenko et al., 2016), and here we showed that this translocation is accompanied by a tendency to an increase of *dfoxo* expression (see Fig. 2). Our data also allow us to assume that *dfoxo* activation under stress results in *dilp6* being inhibited as the decrease of *dilp6* expression found in the control strain w1118 is not observed in *foxo* ^BG01018^ mutants (see Fig. 2).

*dilp6* expression in the fat body was previously shown to suppress dilp2 and *dilp5* expression in imago’s brain and the secretion of DILP2 into hemolymph; *dfoxo*’s influence on the expression of DILPs produced in median neurosecretory cells is inhibited by a simultaneous repression of *dilp6* in the fat body *via*RNA interference (Bai et al., 2012). This allow us to suppose that a decrease in *dilp6* activity under heat stress leads to an increase in level of DILPs expressed in median neurosecretory cells of the brain. Indeed, we were able to demonstrate earlier that DILP3 synthesis in these cells is increased in response to heat stress in wild type flies (Andreenkova et al., 2018), and in *dilp6* ^41^larvae – under normal conditions (Andreenkova et al., 2017), which corresponds well with our assumption about a signal being transmitted from *dfoxo* to DILP3 through *dilp6* under heat stress. Then, DILP3 appears to activate dInR, inhibiting the IIS pathway, which is confirmed by our data on the lack of a shift in dInR expression level under heat stress in flies with mutations of *dilp6* and *dfoxo* genes as opposed to the shift in laboratory strain w1118, in which a decrease in *dilp6* and an increase in dInR expression is shown to occur in response to heat stress (see Fig. 2).

System defects in the IIS pathway cause *D. melanogaster* to manifest a number of different phenotypes including those connected to metabolism, which usually involves an increase in organism’s carbohydrates and lipids reserves (Mattila, Hietakangas, 2017). Murillo-Maldonado et al. (2011) demonstrated almost all viable combinations of mutations with partial loss of function or hypomorphism of IIS genes to have changes in carbohydrates and lipids levels. Slaidina et al. (2009) showed *dilp6* knockdown to cause an increased level of triglycerides and glycogen in Drosophila larvae.These results correspond well with our data on the increased content of total lipids in females of *dilp6* ^41^and *foxo* ^BG01018^ strains (see Fig. 3, a), as well as with increased glucose and trehalose levels in *dilp6* ^41^and *foxo* ^BG01018^ mutants we previously demonstrated (Eremina et al., 2019).

Regarding regulation of feeding behaviour under heat stress it seems to occur independently from *dilp6* and *dfoxo* genes as their mutations do not inhibit loss of appetite following stress (see Fig. 3, b).

## Conclusion

Thus, we have shown that the disruption of *dilp6* and *dfoxo* gene functions in *Drosophila melanogaster* (1) results in the feedback loop of the IIS pathway being disrupted under heat stress, (2) leads to an increase in total lipids content under normal conditions and impedes their decrease following heat stress, and (3) causes an increase in feeding intensity under normal conditions but does not impede its decrease following heat stress.

## Conflict of interest

The authors declare no conflict of interest.
